# Chemical Profiling, Pharmacological Activities, and Toxicity Assessment of *Juniperus phoenicea* sp. *turbinata* L. Extract: Integrated In Vitro, In Vivo, and In Silico Insights

**DOI:** 10.1002/fsn3.71878

**Published:** 2026-05-18

**Authors:** Tarik Chelouati, Soufyane Lafraxo, Amira Metouekel, Mostafa Messkini, Salah‐eddine Chebaibi, Abdelfattah E. L. Moussaoui, Azeddin El Barnossi, Esmael M. Alyami, Ohoud A. Alghamdi, Yousef A. Bin Jardan, Gehan M. Elossaily, Musaab Dauelbait, Musa A. Said, Ahmed Samir Benjelloun

**Affiliations:** ^1^ Laboratory of Biotechnology, Conservation, and Valorization of Bioresources (BCVB), Department of Biology, Faculty of Sciences Dhar El Mahraz Sidi Mohamed Ben Abdellah University Fez Morocco; ^2^ Laboratory of Biotechnology and Environment Sidi Mohammed Ben Abdellah University Fez Morocco; ^3^ Euromed Research Center Euromed University of Fes (UEMF) Fez Morocco; ^4^ Plant Biotechnology Team Abdelmalek Essaadi University Tetouan Morocco; ^5^ Biological Engineering Laboratory Sultan Moulay Slimane University Beni Mellal Morocco; ^6^ Department of Biology King Khalid University Abha Saudi Arabia; ^7^ Health and Medical Research Centre (HMRC) King Khalid University Abha Saudi Arabia; ^8^ Department of Pharmaceutics King Saud University Riyadh Saudi Arabia; ^9^ Department of Basic Medical Sciences AlMaarefa University Diriyah Saudi Arabia; ^10^ Research Center, Deanship of Scientific Research and Post‐Graduate Studies AlMaarefa University Diriyah Saudi Arabia; ^11^ University of Bahr El Ghazal Wau South Sudan; ^12^ Department of Chemistry, Faculty of Science Islamic University of Madinah Madinah Saudi Arabia

**Keywords:** anti‐inflammatory, antimicrobial, antioxidant, in silico, *Juniperus*, LC–MS/MS

## Abstract

*Juniperus phoenicea* ssp. *Turbinata* L. ( *J. phoenicea* ssp. *Turbinata)* has long been used in traditional medicine to treat a variety of ailments. This study aimed to investigate the pharmacological potential and to characterize the phytochemical composition of the hydroethanolic leaf (JPEF) and fruit (JPEG) extracts. Liquid Chromatography Tandem Mass Spectrometry (LC–MS/MS) analysis revealed that both JPEF and JPEG contained high levels of phenolic compounds, with ferulic acid identified as the dominant constituent. The biological evaluation began with acute toxicity tests in rodents, which indicated that JPEF and JPEG extracts were well tolerated at the doses administered, with no significant adverse effects observed. The extracts exhibited strong anti‐inflammatory activity (94.23% for JPEF; 83.24% for JPEG), comparable to that of diclofenac (97.02%), as well as a notable analgesic effect (75% and 70.62%), compared to tramadol (87.01%). Significant antioxidant activity was observed (DPPH IC_50_: 70.2 and 90.76 μg/mL), accompanied by antimicrobial activity (bacterial inhibition zones: 11.44–20.36 mm; fungal: 13.59–20.38 mm). Molecular docking revealed a stable interaction between the compounds and several enzymatic targets. These results confirm the potential of *J. phoenicea* ssp. turbinata as a promising source of therapeutic agents.

## Graphical abstract

Hydroethanolic leaf and fruit extracts of *Juniperus phoenicea* showed high phenolic content, dominated by ferulic acid. Both extracts were non‐toxic and exhibited strong anti‐inflammatory, analgesic, antioxidant, and antimicrobial activities. Molecular docking confirmed stable binding to key inflammatory and microbial targets, supporting its pharmaceutical potential.

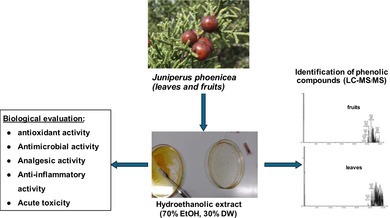

## 1 Introduction

Medicinal plants have been central to traditional healthcare systems since ancient times. The World Health Organization (WHO) estimates that more than 85% of traditional medical knowledge worldwide is based on plant extracts (WHO 2004). For this reason, medicinal plants have been used for centuries to treat a variety of illnesses. Their abundance of bioactive molecules with medicinal properties makes them an untapped reservoir of potential pharmaceuticals. Antioxidant compounds derived from plant extracts may provide a means of treating ailments such as oxidative stress‐induced inflammation (Arulselvan et al. 2016). The body uses inflammation as a defense mechanism, but it can occasionally cause diseases that are treated with traditional anti‐inflammatory medications (steroidal and non‐steroidal). These compounds work well, but their use may be limited due to frequent adverse effects on the digestive and cardiovascular systems (Faridi et al. 2014). In this regard, the use of natural resources, particularly medicinal plants, is emerging as a viable alternative to potent medications with fewer adverse effects (Chebaibi et al. 2011).

In North Africa's traditional folk pharmacopeia, *J. phoenicea* ssp. *turbinata*, also known as “Araar,” is a member of the Cupressaceae family and one of the most widely used herbs (Benkhnigue et al. 2014; Benlamdini et al. 2014; Lahsissene et al. 2009). The tree's various parts are used in medicinal preparations for antioxidant and antimicrobial (Chelouati et al. 2023, 2024), antidiarrheal (Dob et al. 2008; Mazari et al. 2010), and antiseptic effects (Medini et al. 2007; Stassi et al. 1996). These components are used to treat conditions including rheumatism and diabetes (Allali et al. 2008; Stassi et al. 1996). Berries, twigs, and young shoots, when prepared as infusions, are stomachic and digestive (Barrero et al. 2004; Bellakhdar 1997). Skin abscesses and ulcers can be treated with dried and powdered berries (Akrout 2004; Qnais et al. 2005). A leaf‐berry combination from this plant also has hypoglycemic properties (Amer et al. 1994). To lend scientific credibility to traditional claims for their use in various treatments, the present study assessed the toxicity and previously unidentified anti‐inflammatory and analgesic properties of JPEF and JPEG using in vivo and in silico assays. The study also aimed to investigate antioxidant and antimicrobial activity in vitro and in silico, and to use LC–MS/MS to characterize the chemical composition of the JPEF and JPEG.

## 2 Materials and Methods

### 2.1 Plant Material and Extract Preparation

#### 2.1.1 Identification of the Plant

*Juniperus phoenicea turbinate* (L.) was manually picked in March 2024 from a mountainous population in the rural commune Dar Al Hamra, Sefrou area (33.75750029; −4.32896628). Professor Bari Amina identified the specimens, which were deposited in the herbarium of the Faculty of Sciences at Dhar El Mahraz University, Sidi Mohamed Ben Abdellah, Fez, Morocco. under voucher number LSNA/TC/JP‐24.

#### 2.1.2 Preparation of JPEF and JPEG

10 g of fruits and leaves were pulverized into a uniform powder. 10 g of this powder was combined with 30 mL of distilled water and 70 mL of ethanol for extraction at 70% (v/v). It is a 1:10 plant/solvent ratio. This combination was allowed to macerate at room temperature (26°C) for 3 days, with occasional agitation. Following that, Whatman number 4 paper was used to filter the mixture. The resulting filtrate was dried and concentrated in an oven at 40°C in the dark until a dry powder was obtained. The resulting dry JPEF and JPEG were kept in stained vials at 4°C. To ensure that the results could be replicated, each extraction was carried out three times (Bouslamti et al. 2022).

#### 2.1.3 Yield Determination

By comparing the initial mass of the plant material utilized (in grams) with the mass of the extract (Lafraxo et al. 2023), we may determine the yield. The yield is expressed as a % using the formula below:

(1) $$\text{Yield}\;\left(\%\right)=\frac{\text{weight of dried extract}\left(g\right)}{\text{weight of plant material}\left(g\right)}\times 100$$

### 2.2 LC–MS/MS Analysis

Using the LC–MS/MS method, the phytochemical analysis of JPEG and JPEF was completed. The materials were meticulously filtered via a Millex‐GP disposable syringe filter disc with a 0.45 μm pore size before being injected. We employed the ThermoFisher Scientific DIONEX UltiMate 3000 LC–MS/MS Extractive Plus system (UHPLC+) for chromatography, which was connected to an electrospray ionization mass spectrometer (ESI‐MS/MS) and a diode array detector (DAD). A high‐resolution quadrupole‐OrbitrapTM mass detector encompassing a mass range of 50 to 6000 m/z was used. Thermo Scientific Chromeleon (version 4.1, Thermo Xcalibur Roadmap) software was used to analyze the generated chromatograms. Using a Thermo ScientificTM HypersilTM BDS C18 column (150 mm, 4 mm × 6 mm, 5 μm), chromatographic separation was carried out. Using an electrospray ionization (ESI) source equipped with a Thermo ScientificTM ExactiveTM Plus Orbitrap mass spectrometer, data acquisition in negative mass detection mode was carried out. For chromatography, the mobile phases were (A) methanol (MeOH) and (B) water acidified with citric acid (pH adjusted to 3.1). Using mobile phases designed to segregate the molecules based on their polarity, the analysis was conducted in gradient mode for 45 min. Compounds were identified by comparing the observed m/z values, retention times, and fragmentation profiles with the MS‐library and MS/MS‐library databases, as well as with data from the literature (Metouekel et al. 2024).

### 2.3 Evaluation of the in Vivo Biological Activity of the JPEF and JPEG Studied

#### 2.3.1 Acute Toxicity Test

##### 2.3.1.1 Animal Used

For this study, Swiss albino mice from the department of Dhar El Mahraz Faculty were used, weighing between 22 and 25 g and aged 8 weeks, from the College of Science in Fez. The mice were housed in standard cages (five per cage) under controlled conditions, with a temperature of 24°C ± 2°C and a 12‐h light–dark cycle. A two‐week acclimation period was observed before the experiments began. Throughout the study, the animals received standard pellets and had free access to water (Jawhari et al. 2021).

##### 2.3.1.2 Preparation of Solutions

To determine the volume of solution to be administered, a common approach is to use a mathematical formula that accounts for factors such as the extract concentration and the animal's body weight (Moussaoui et al. 2020). The following is a general example of a formula (2) that might be used:

(2) V=D×P/C

where V is the volume of solution to be administered in mL; C is the concentration of extract in mg/mL.

##### 2.3.1.3 Acute Toxicity

Acute oral toxicity was assessed according to the guidelines of the Organization for Economic Co‐operation and Development (OECD‐423) (OECD 2001). Mice in experimental groups were given concentrations of each plant extracted with a maximum of 2000 mg/kg. The mice were fasted overnight with free access to water. The mice used were divided into groups. Each group contained five mice of similar weight. Mice in different groups were treated as follows:

Lot I (Control): received a 0.9% NaCl solution at a dose of 10 mL/kg.

Lot II (Treated): received the extract at different maximum doses:

- Lot II‐A: receives the extract at the maximum dose of 200 mg/kg.
- Lot II‐B: receives the extract at a maximum dose of 500 mg/kg.
- Lot II‐C: receives the extract at a maximum dose of 1000 mg/kg.
- Lot II‐D: receives the extract at a maximum dose of 2000 mg/kg.

The mice were observed for 48 h after administration of the JPEF and JPEG. Observations were made of general behavioral changes, signs of toxicity, and mortality rate. Particular attention was paid during the first 4 h after administration of the JPEF and JPEG to detect any immediate effects. For a comprehensive evaluation of the toxicity of the JPEF and JPEG, the body weight of each mouse was measured daily (before and after treatment). Euthanasia was performed by an overdose of sodium pentobarbital (150 mg/kg, intraperitoneally) in accordance with international guidelines (Underwood and Raymond 2013). After sacrifice, organs such as the liver, spleen, and kidneys were carefully removed and individually weighed for further analysis. The relative organ weights of each mouse were calculated:

(3) $$\mathrm{RWO}=\frac{\mathrm{AOW}}{\mathrm{BWS}}\times 100$$

where RWO, relative weight of organs; AOW, absolute organ weight (g); BWS, body weight of the mouse on the day of sacrifice (g).

#### 2.3.2 Anti‐Inflammatory Activity

##### 2.3.2.1 Carrageenan Test

Using Winter's approach (Winter et al. 1962), the anti‐inflammatory efficacy of JPEF and JPEG was evaluated. Ninety minutes before injecting 1% carrageenan dissolved in 0.9% NaCl under the plantar fascia of the rat's right hind paw, the extract was given orally at a concentration of 200 and 400 mg/kg. Before the carrageenan injection, the circumference of the paw was measured, and from the third to the sixth hour following carrageenan administration, it was measured every hour. Diclofenac 1%, a reference anti‐inflammatory, was used as a positive control and administered orally. The rats were divided into different batches, each batch containing 5 rats of similar weight. Diclofenac, a non‐steroidal anti‐inflammatory drug, was used as a reference at a dose of 10 mg/kg. Treatments of the different batches were performed as follows (Lafraxo et al. 2024, Mobashar et al. 2025).

- Lot I (Negative control): receives NaCl (0.9%) solution at a dose of 10 mL/kg.
- Lot II (Reference): received diclofenac solution at a dose of 10 mg/kg.
- Lot III (Treated): received the extract at doses of 200 and 400 mg/kg.

The % of inhibition of inflammation was then calculated according to the following formula:

(4) $$\text{Inhibition}\;\left(\%\right)=⟮1-\frac{\left(\mathrm{St}-S0\right)\;\text{Treated}\;}{\left(\mathrm{St}-S0\right)\text{control}}⟯\times 100$$

where S0 is the circumference of the paw before carrageenan injection, and St is the circumference of the paw at a given time after carrageenan administration.

##### 2.3.2.2 Analgesic Test

The principle of this test is to induce a painful syndrome in rats by injecting acetic acid, resulting in contortions and stretching movements (El Moussaoui et al. 2020).

The rats, divided into 4 lots, were treated as follows:

- Lot I (Control): received a solution of NaCl (0.9%) at a dose of 10 mL/kg.
- Lot II (Reference): received a solution of Tramadol at 10 mg/kg
- Lot III and IV (Treated) received the extract at doses of 200 and 400 mg/kg, respectively.

The rats were given an intraperitoneal injection of a 0.6% acetic acid solution at a rate of 10 mL/kg, 30 min after undergoing these various treatments. Each rat was put in an observation cage as soon as the acetic acid injection was given, and the number of contortions was counted for 15 min. For every treatment group, the % of inhibition of acetic acid‐induced contortions was computed using the following formula:

(5) $$\text{Inhibition}\;\left(\%\right)=\frac{C-T}{C}\times 100$$

where C is the mean number of contortions in the control group, and T is the mean number of contortions in the treated group (extract or reference).

### 2.4 Evaluation of the In Vitro Biological Activity of the JPEF and JPEG Studied

#### 2.4.1 Antioxidant Power

##### 2.4.1.1 DPPH Test

A volume of 25 μL of each methanolic extract solution at various concentrations was added to 975 μL of DPPH methanolic solution (2.4 mg/100 mL methanol) to assess the antioxidant activity of JPEF and JPEG. A negative control was created by combining 975 μL of the DPPH methanolic solution with 25 μL of methanol. The tubes were vortexed and then left at room temperature for half an hour in the dark. At 517 nm, absorbance was measured. The following formula can be used to express the results as free radical inhibition (I%) or free radical scavenging activity in % (Tepe et al. 2005)

(6) $$\begin{array}{cc} \text{Inhibition}\;\left(\%\right)\;=\; & \left[\left(\mathrm{Abs}\;\text{Negative Control}-\mathrm{Abs}\;\text{Sample}\right)\right]\\ & /\mathrm{Abs}\;\text{Negative Control}\times 100 \end{array}$$

where %: Percentage of anti‐free radical activity; Abs Sample: Absorbance of the sample after 30 min; Abs Negative Control: Absorbance of the negative control after 30 min. To better express the results of the anti‐free radical effect of the JPEF and JPEG, three parameters were calculated.

##### 2.4.1.2 FRAP Test

To determine the reducing power of methanolic JPEF and JPEG, we used the Oyaizu method (Oyaizu 1986). To tubes containing 1 mL of solutions of JPEF and JPEG at different concentrations, we added 2.5 mL of a 0.2 M phosphate buffer solution (pH 6.6) and 2.5 mL of a 1% potassium ferricyanide K_3_Fe(CN)_6_ solution. The mixtures were incubated at 50°C for 20 min. Next, 2.5 mL of 10% trichloroacetic acid was added to block the reaction. Next, 2.5 mL of the reaction mixture was mixed with 2.5 mL of distilled water and 0.5 mL of a freshly prepared 0.1% aqueous FeCl_3_ solution. The absorbance of the reaction medium was measured at 700 nm. The EC_50_ values, representing the concentration required to achieve 50% inhibition, were determined from the dose–response curves. The results were expressed as absorbance values and as EC_50_ values (μg/mL).

##### 2.4.1.3 TAC Test

One milliliter of liquid reactive solution (0.6 M sulfuric acid, 28 mM sodium phosphate, and 4 mM ammonium molybdate) was combined with 25 μL of each extract under study (Maškovic et al. 2012). Following a 90‐min incubation period at 95°C, the optical density was assessed using a spectrophotometer set to 695 nm and a blank that contained 25 μL of methanol in place of the extract. Using an ascorbic acid standard curve, the antioxidant capacity was reported in milligrams of ascorbic acid equivalent per gram of JPEF and JPEG (μg EAA/mg extracts).

#### 2.4.2 Antimicrobial Activity

The antimicrobial activity of JPEF and JPEG was assessed by testing the following microbial strains: *Escherichia coli* ATCC 29213, *Klebsiella pneumoniae* CIPA22, *Staphylococcus aureus* ATCC 6633, *Proteus mirabilis* ATCC 29906, *Candida albicans* FMP19/F and *Saccharomyces cerevisiae* FMP18/F. JPEF and JPEG were prepared with dimethyl sulphoxide (DMSO, 3%), then serially diluted from 1/2 to 1/64. Each strain was inoculated onto nutrient agar to obtain isolated colonies. After incubation for 24 h at 37°C, 4–5 well‐isolated colonies were picked and suspended in sterile distilled water to obtain a cell density equivalent to 0.5 McFarland (≈10^6^ CFU/mL) (El Barnossi et al. 2020, 2021). The bacterial inoculum was evenly spread onto Mueller‐Hinton agar plates, while the fungal inoculum was spread onto Sabouraud dextrose agar plates using a sterile swab, rotating the plate at 60° after each application to ensure uniform distribution. Sterile discs impregnated with 10 μL of extract at different concentrations were placed on the agar, with control discs soaked in DMSO as negative controls. Plates were incubated at 37°C for 24 h for bacteria and 48 h for yeast. Antimicrobial activity was determined by measuring the diameters of the zones of inhibition around the discs.

### 2.5 In Silico Evaluation of Biological Activity

#### 2.5.1 Ligand Preparation

The docking study focused on phytocompounds identified in JPEF and JPEG. These molecules were obtained in SDF format from the PubChem database and prepared using the LigPrep module in Maestro (version 11.5, Schrödinger). The OPLS3 force field was applied for 3D optimization and energy minimization. To reflect physiological conditions, ionization states were adjusted to pH 7.0 ± 2.0. LigPrep was also configured to generate up to 32 stereoisomers per compound to account for potential stereochemical influences on binding interactions (Chebaibi et al. 2024).

#### 2.5.2 Protein Target Selection and Preparation

To comprehensively evaluate the multi‐pharmacological profile of the compounds under investigation, four key protein targets were meticulously selected from the RCSB Protein Data Bank (www.rcsb.org). Their selection was based on their established biological relevance to the targeted therapeutic activities. The specific objectives for molecular docking against each target are detailed in Table 1.

**TABLE 1:** Characteristics of protein targets and biological justifications for docking studies.

Biological activity	Protein target (PDB ID)	Justification for target selection	References
Antioxidant	NADPH oxidase (2CDU)	A major enzyme responsible for the production of reactive oxygen species (ROS); its inhibition is a key mechanism to counteract oxidative stress	(Drummond et al. 2011; Mhya et al. 2023)
Antibacterial	DNA Gyrase B (3G7E)	A type II topoisomerase essential for bacterial DNA replication; a validated target for the development of novel antibacterial agents	(Alotaibi et al. 2024; Leng et al. 2022)
Antifungal	Ergosterol (5FSA)	A vital structural component of fungal cell membranes; interaction with this target or its biosynthesis enzymes is crucial for antifungal activity	(Barrett‐Bee and Dixon 1995; Wong‐Deyrup et al. 2020)
Anti‐inflammatory	5‐Lipoxygenase (3 V99)	An enzyme initiating the biosynthesis of leukotrienes, which are major pro‐inflammatory mediators; its inhibition reduces the inflammatory response	(Lončarić et al. 2020; Tsolaki et al. 2018)

Each protein structure underwent preparation using the Protein Preparation Wizard within the Maestro software suite (Schrödinger LLC). The preparation steps included the addition of missing hydrogen atoms, correction of bond orders, and removal of crystallographic water molecules located beyond 5 Å from the active site. Water molecules deemed essential for active site stabilization were retained. Titratable residues (e.g., histidine, glutamate, aspartate) were protonated according to pKa predictions to mimic physiological pH. Finally, restrained energy minimization was performed using the OPLS3 force field (Harder et al. 2016) to optimize structural geometry while maintaining consistency with experimental data (Beniaich et al. 2023).

#### 2.5.3 Glide Standard Precision (SP) Ligand Docking

Docking was performed using the Glide SP module in Maestro. Receptor grids were centered on the co‐crystallized ligand or known active site residues. Ligands were docked flexibly to sample multiple orientations, and poses were scored based on binding affinity and geometric fit. The conformation with the lowest Glide score was selected as the optimal binding mode for each ligand (Slighoua et al. 2023).

### 2.6 Statistical Analysis

The experimental data are expressed as mean ± standard deviation. Statistical analyses were performed using GraphPad Prism software, version 8.0. The normality of the data was tested using the Shapiro–Wilk test, and the results showed that all data followed a normal distribution (*p* > 0.05). The homogeneity of variances was assessed using Levene's test, which indicated that the variances were not homogeneous across the groups (*p* < 0.05). A one‐way analysis of variance (ANOVA) was used to assess differences between groups. Where the ANOVA indicated statistically significant differences (*p* < 0.05), Tukey's multiple comparison test was performed as a post hoc analysis to identify pairwise differences between groups. A *p*‐value of less than 0.05 was considered statistically significant.

## 3 Results and Discussion

### 3.1 Phytochemical Identification by LC–MS/MS

**FIGURE 1:**
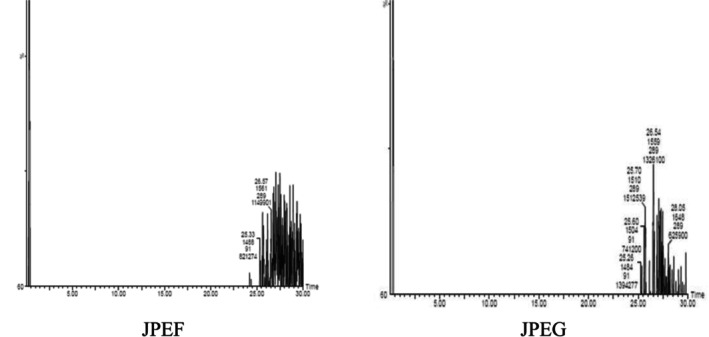
Chromatographic profile of JPEF and JPEG extracts studied.

The extraction method used gave a yield of 7.48% ± 0.81% (w/w, *n* = 3) for JPEF and 6.92% ± 0.73% (w/w, *n* = 3) for JPEG based on the dry weight of the plant material. These results are significant compared to those obtained for *Juniperus communis* and *Juniperus drupacea* , with percentages of 6.40% and 6.65%, respectively (Taviano et al. 2011). According to Dane et al. (2016), variations in extraction yields can be attributed to the bioavailability of extractable compounds, which in turn depends on the chemical composition of the plants (Figure 1).

Phytochemical characterization of the JPEF and JPEG studied by LC–MS/MS revealed the presence of certain phytochemical compounds. Based on the retention time of the standards, the *J. phoenicea* ssp. *turbinata* extract showed a chemical profile comprising nine phenolic compounds identified in this medicinal plant, with a dominance of dihydroferulic acid 4‐O‐glucuronide in JPEG and ferulic acid 4‐O‐glucuronide in JPEF (Table 2).

**TABLE 2:** Tentative identification of anthocyanin and phenolic compounds by LC–MS/MS (*n* = 3).

No.	RT (min)	m/z (M‐H)	Fragments	Proposed compounds	JPEF (%)	JPEG (%)
1	0.25	371.08	209.12/191.23	Dihydroferulic acid 4‐O‐glucuronide	—	35.27
2	0.31	369.94	289.14/191.23	Ferulic acid‐4‐O‐glucuronide	32.22	—
3	25.27	373.87	289.07/113.35	7‐Hydroxymatairesinol	6.33	4.47
4	25.33	610.02	541.27/237.32	Cyanidin‐3,5‐O‐diglucoside	12.25	5.85
5	25.7	630.12	541.27	Pelargonidin‐3,5‐O‐diglucoside	15.19	11.12
6	26.1	515.5	131/91	4,5‐O‐dicaffeoylquinic acid	12.11	8.25
7	26.5	325.25	163.1	p‐Coumaric acid 4‐O‐glucoside	8.65	18.14
8	26.8	577.8	290	Procyanidin dimer B1	6.14	6.34
9	27.2	577.8	290	Procyanidin dimer B1 isomer 1	3.14	2.81
10	28.8	511.12	—	nd	1.12	2.10

### 3.2 Acute Toxicity

**FIGURE 2:**
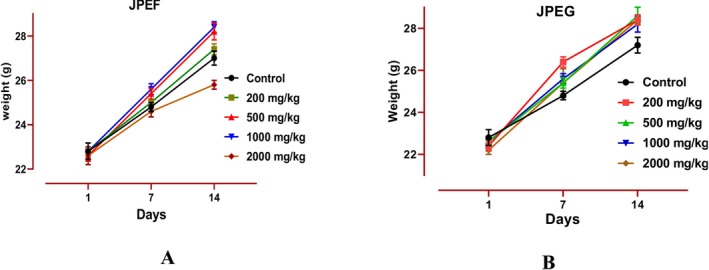
Effect of JPEF (A) and JFEG (B) on body weight of experimental animals during the treatment period. With *n* = 5, the data are presented as mean value ± SD.

After administration of the JPEF and JPEG to the mice, they were observed for 24 h to record clinical symptoms. During this period, no clinical symptoms appeared in the mice treated (diarrhea, immobility, excitement, contortion, refusal of food, tremors, and mortality). The body weight and general behaviors of the animals were frequently assessed to detect the appearance or absence of a toxic effect (Sireeratawong et al. 2008). Oral administration of *J. phoenicea* ssp. *Turbinata* leaf extract (JPEF) to mice, up to a dose of 1000 mg/kg, under conditions of acute toxicity, had no adverse effect on the animals' behavior or body weight. However, at a dose of 2000 mg/kg, a slight decrease in body weight was observed 14 days after administration compared to the control group, with respective body weights of 25.80 and 27.0 g (Figure 2A). This difference was not statistically significant. Although no mortality was observed, this observation suggests that the high dose of 2000 mg/kg may be slightly toxic to the health of mice. Oral administration of JPEG to mice, up to a maximum dose of 2000 mg/kg, under conditions of acute toxicity, had no adverse effect on the animals' behavior or body weight. Compared with the control group, all mice given the JPEG extract showed an increase in body weight over a period of 14 days. This indicates that the JPEG is not toxic to the health of the mice (Figure 2B).

**FIGURE 3:**
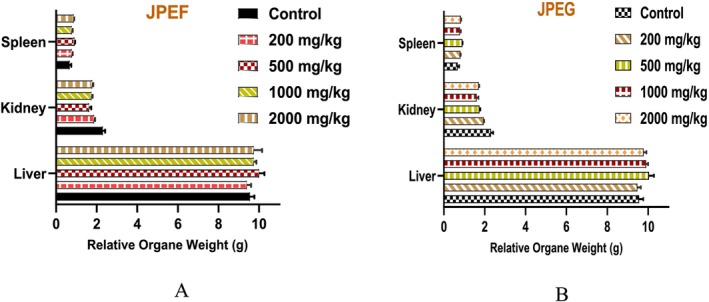
Relative weight of animal organs administered by JPEF (A) and JPEG (B). With *n* = 5, the data are presented as mean value ± SD.

After 14 days of administration of the extract, the mice were sacrificed to allow the collection and weighing of organs such as the liver, kidneys, and spleen (Figure 3). The relative weight of these organs was calculated. It was found that there was no significant difference in the relative weight of the kidneys, liver, and spleen of the treated mice compared with the control group. These results corroborate previous studies showing that the administration of toxic substances, whether natural or chemical, can reduce the weight of internal organs (Raza et al. 2002). We can therefore conclude that the extract tested is safe for animals at doses of up to 2000 mg/kg/day.

JPEF and JPEG presented no significant risk. In particular, JPEF was found to be less toxic than JPEG (El‐Sawi et al. 2014). In addition, ethanolic extracts of *J. communis* berries were well tolerated and showed no side effects in rats at a dose of 2500 mg/kg. Similarly, extracts of *J. communis* stems administered intraperitoneally at a dose of 2000 mg/kg showed no toxicity (Garg 2010).

Changes in body weight are used as a general indicator of the adverse effects of JPEF and JPEG testing, reflecting animal welfare (Unuofin et al. 2018). Surviving animals should not lose more than 10% of their initial body weight. Weight loss is correlated with the physiological state of the animal, such as metabolism, and can be attributed to an anorexic effect (Betti et al. 2012). It has been reported that oral gavage can lead to numerous complications, increasing the likelihood of mortality, including esophageal perforation, aspiration pneumonia, and stress (Park et al. 2019).

Organ weights are essential indicators of the physiological and pathological state of animals. Variation in organ weights is a specific sign of toxicity in laboratory animals. The toxic effect of herbal remedies is most likely to occur in vital organs such as the spleen, liver, and kidneys, due to their crucial role in the body. The liver is the main organ for the biotransformation of xenobiotics, while the kidneys are responsible for the excretion of these substances (Unuofin et al. 2018). Finally, we did not determine the LD_50_ because our objective was to evaluate the safety and tolerability of the doses commonly used for pharmacological testing, rather than establishing the lethal dose parameter.

### 3.3 Anti‐Inflammatory Activity

**FIGURE 4:**
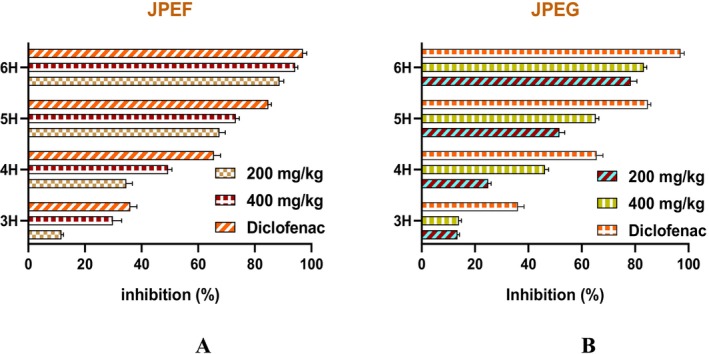
Anti‐inflammatory power of JPEF (A) and JPEG (B) by the carrageenan method. With *n* = 5, the data are presented as mean value ± SD.

The anti‐inflammatory activity of the JPEF and JPEG studied was assessed using the carrageenan method. The results of this activity are shown in Figure 4. The leaf extract showed inhibition of 11.70%, 29.86%, and 36.05% after 3 h of treatment with doses of 200, 400 mg/kg, and diclofenac, respectively. After 6 h of treatment with JPEF (200 and 400 mg/kg) and positive control (Diclofenac), the anti‐inflammatory potency was 88.77%, 94.23%, and 97.02%, respectively (Figure 4A). On the other hand, treatment for 3 h with JPEG (200 and 400 mg/kg) revealed inhibition of 13.43% and 13.94%, respectively. After 6 h of treatment with JPEG (200 and 400 mg/kg), inhibition was 78.39% and 83.24%, respectively (Figure 4B). The doses tested for both JPEF and JPEG showed that 400 mg/kg was more effective than 200 mg/kg, with maximum efficacy recorded for the positive control (diclofenac). This control is a cyclooxygenase inhibitor and is one of the anti‐inflammatory agents most commonly used as a control in the search for new anti‐inflammatory drugs (Kim et al. 2008).

**FIGURE 5:**
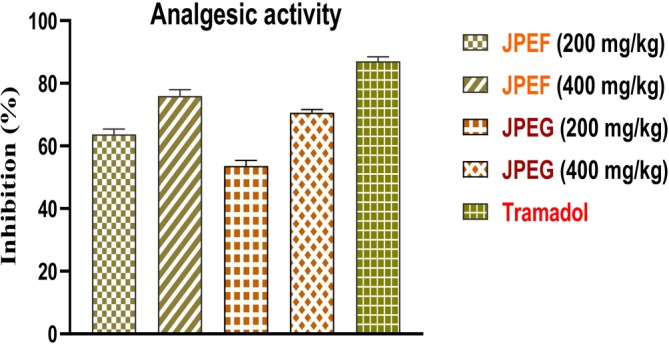
Analgesic effect of JPEF, JPEG, and positive control (Tramadol). With *n* = 5, the data are presented as mean value ± SD.

The analgesic activity was evaluated as a complementary measure of anti‐inflammatory potential. In this model, acetic acid induces inflammatory pain by stimulating the release of endogenous mediators such as histamine, prostaglandins (PGE2 and PGEα), serotonin, bradykinin, substance P, and cytokines such as IL‐1β, TNF‐α, and IL‐8 (Banerjee et al. 2012; Florentino et al. 2016; Ribeiro et al. 2010). The results of the analgesic activity revealed that the leaf extract, at doses of 200 and 400 mg/kg, showed inhibitions of the order of 63.74% and 75%, respectively (Figure 5). In comparison, doses of 200 and 400 mg/kg of fruit extract showed inhibitions of the order of 53.63% and 70.62%, respectively. These results indicate that the leaf extract is more effective than the fruit extract, with maximum efficacy recorded for the positive control (Tramadol), which showed inhibition of the order of 87.01%.

Inflammation is an immunological defense mechanism triggered in response to various noxious stimuli (Aouey et al. 2016). It primarily occurs in three separate stages. An increase in vascular permeability that causes blood fluids to exude into the interstitial space characterizes the first phase. Leukocytes from the blood infiltrate the tissues during the second phase. Ultimately, the development of granulomas and tissue healing characterizes the third phase (Bagad et al. 2013).

Natural products, particularly those derived from higher plants, have long been a valuable source of substances with therapeutic potential, despite limited knowledge of their mechanisms of action (Igbe et al. 2010; Jan and Khan 2016). Identifying molecules capable of inhibiting neutrophil infiltration into inflammatory sites could offer new avenues for the treatment of inflammatory diseases. According to Kouakou et al. (2010), flavonoids have anti‐inflammatory properties by inhibiting the production of prostaglandins and leukotrienes. It has been reported that flavonoids such as quercetin, kaempferol, isorhamnetin, rutin, luteolin, hesperidin, and biflavonoids, as well as terpenes, coumarins, tannins, and steroids, are responsible for significant anti‐nociceptive and/or anti‐inflammatory effects (Alwashli et al. 2012; Hosseinzadeh and Younesi 2002; Ojewole 2007). Several studies have shown the role of flavonoids and tannins isolated from medicinal plants as analgesic and anti‐inflammatory agents (Afsar et al. 2015; Hossain et al. 2014). For example, rutin and caffeic acid have an inhibitory effect on COX and 5‐lipoxygenase (Afsar et al. 2015). The presence of phenolic compounds revealed by LC–MS/MS could explain the anti‐inflammatory activity observed in these tested JPEF and JPEG.

### 3.4 Antioxidant Power

**FIGURE 6:**
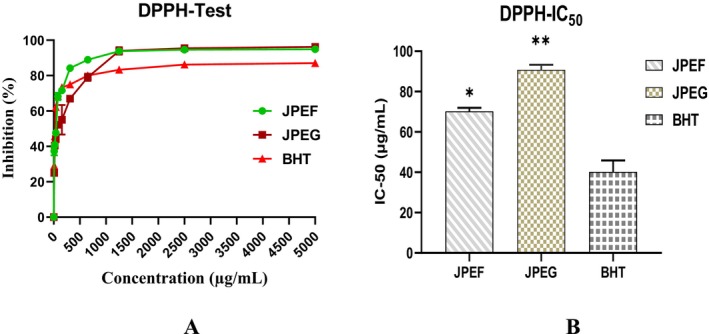
Antioxidant capacity of JPEF and JPEG determined by the DPPH method (A) and IC_50_ (B) (mean ± SD). *Comparison between the extract groups and the control group; **p < 0.01; *p < 0.05.

The results of the antioxidant power of JPEF and JPEG, measured by the DPPH method, showed that as the concentration increased, the % of inhibition also increased. At a concentration of 10 μg/mL, JPEF and JPEG showed 37.27% and 25.07% inhibition, respectively. On the other hand, at a high concentration of 300 μg/mL, JPEF and JPEG showed 84.24% and 66.96% inhibition, respectively (Figure 6A).

Antioxidant efficacy was evaluated using the DPPH test by determining the concentration required to inhibit 50% of DPPH free radicals (IC_50_). The results showed IC_50_ values of 70.2 μg/mL for JPEF, 90.76 μg/mL for JPEG, and 40.22 μg/mL for the positive control BHT. Since lower IC_50_ values indicate stronger antiradical activity, BHT exhibited the highest antioxidant capacity, followed by JPEF, while JPEG showed relatively lower activity (Figure 6B).

**FIGURE 7:**
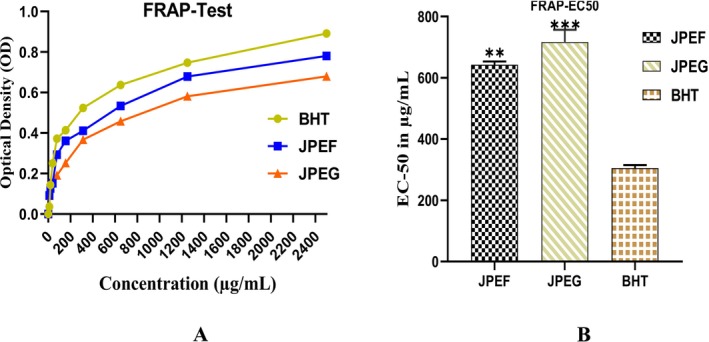
Antioxidant capacity of JPEF and JPEG determined by the FRAP method (A) and EC_50_ (B) (mean ± SD). *Comparison between the extract groups and the control group; **p < 0.01; ***p < 0.001.

The second test to evaluate antioxidant power was carried out using the *Ferric Reducing Antioxidant Power* (FRAP) method. The results of this test showed that the antioxidant effect increased with the concentration of the JPEF and JPEG studied, as indicated by an increase in optical density (Figure 7A). At a concentration of 20 μg/mL, JPEF and JPEG revealed an optical density of 0.144 and 0.126, respectively. On the other hand, at a concentration of 150 μg/mL, JPEF and JPEG revealed an optical density of 0.415 and 0.361, respectively. Finally, at a high concentration of 650 μg/mL, JPEF and JPEG showed optical densities of 0.637 and 0.534, respectively. To compare the antioxidant efficiency of the FRAP method, it is necessary to measure the effective concentration (EC_50_). Evaluation of antioxidant power by this method revealed an EC_50_ of 642.5 μg/mL for JPEF, 717 μg/mL for JPEG, and 305 μg/mL for the positive control (BHT) (Figure 7B).

**FIGURE 8:**
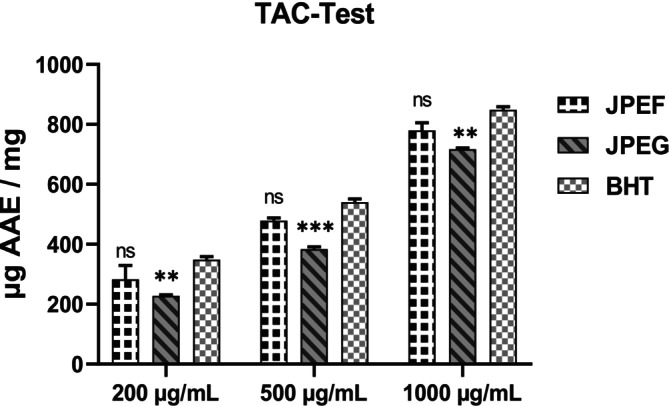
Antioxidant capacity of JPEF, JPEG, and BHT studied by the phosphomolybdate (TAC) method (mean ± SD). *Comparison between the extract groups and the control group; **p < 0.01; ***p < 0.001; ns: Non‐significant.

The third method for assessing the antioxidant capacity of the JPEF and JPEG studied was the phosphomolybdate method. At a concentration of 200 μg/mL, the results showed a total antioxidant capacity of 283 μg AAE/mg for JPEF, 227.5 μg AAE/mg for JPEG, and 349 μg AAE/mg for BHT (Figure 8). At a concentration of 500 μg/mL, the antioxidant power was 478.5 μg EAA/mg for JPEF, 383.5 μg EAA/mg for JPEG, and 540 μg EAA/mg for BHT. Finally, at a higher concentration of 1000 μg/mL, the total antioxidant capacity was 779.5 μg EAA/mg for JPEF, 717 μg EAA/mg for JPEG, and 848.5 μg EAA/mg for BHT (Figure 8).

The presence of phenolic compounds detected in the extract is the main reason for the antioxidant activity demonstrated in this experiment. Phenolics are known to neutralize free radicals primarily through hydrogen atom transfer (HAT) or single electron transfer (SET), in which a hydrogen atom is contributed to DPPH• to generate the stable DPPH–H molecule (Popovici et al. 2009). The ability of phenolics obtained from plants to scavenge free radicals is believed to originate mostly from this mechanism. Previous research has demonstrated the importance of several phenolic acids in the antioxidant capacity of natural products, including quercetin, rutin, caffeic acid, and p‐coumaric acid (Ali et al. 2010).

In our LC–MS/MS profile, compounds such as dihydroferulic acid 4‐O‐glucuronide, ferulic acid‐4‐O‐glucuronide, and 4,5‐O‐dicaffeoylquinic acid are of particular interest because derivatives of ferulic and caffeic acids are well‐documented for their strong radical‐scavenging abilities due to their conjugated aromatic systems and hydroxyl substitutions, which stabilize phenoxyl radicals. Additionally, the detection of pelargonidin‐3,5‐O‐diglucoside and cyanidin‐3,5‐O‐diglucoside supports the measured antioxidant activity. Anthocyanins, including cyanidin and pelargonidin derivatives, are highly efficient hydrogen donors and possess strong electron‐delocalization capacities, which enhance their antioxidant behavior. The presence of 7‐hydroxymatairesinol, a lignan with established redox properties, may also contribute synergistically to the overall activity.

Generally, ferulic acid and its derivatives are recognized for their ability to trap free radicals through the donation of hydrogen atoms and resonance stabilization of the phenoxyl radicals formed. This structural characteristic enhances their effectiveness in neutralizing reactive oxygen species and contributes significantly to the free radical scavenging activity observed in this study (Jacobo‐Velázquez 2025).

Numerous biological actions, such as antioxidant, antibacterial, anti‐inflammatory, anticancer, hepatoprotective, vasoprotective, neuroprotective, cardioprotective, and antidiabetic effects, have been reported for phenolic compounds, especially flavonoids and related structures (Tavares and Seca 2018; Younis et al. 2016). However, it can be difficult to accurately predict each molecule's antioxidant action in complex mixtures due to the structural complexity and chemical diversity within phenolic groups (Djeridane et al. 2006). Furthermore, there may not always be a perfect correlation between antioxidant tests and the total phenolic content (TPC), which may not include all of the antioxidants in the extract. Variations in redox potential across compounds, fluctuating molar absorptivity in colorimetric tests, and the antagonistic or synergistic interactions among phenolics in the mixture are some of the reasons for this disparity (Almela et al. 2006; Djeridane et al. 2006).

Overall, the study's antioxidant capacity is in line with the LC–MS/MS‐revealed qualitative and quantitative phenolic profile. The extract's total capacity to scavenge radicals is probably influenced by the presence of phenolic acids, anthocyanins, and lignans, each of which has unique but complementary redox properties.

### 3.5 Antimicrobial Properties

**FIGURE 9:**
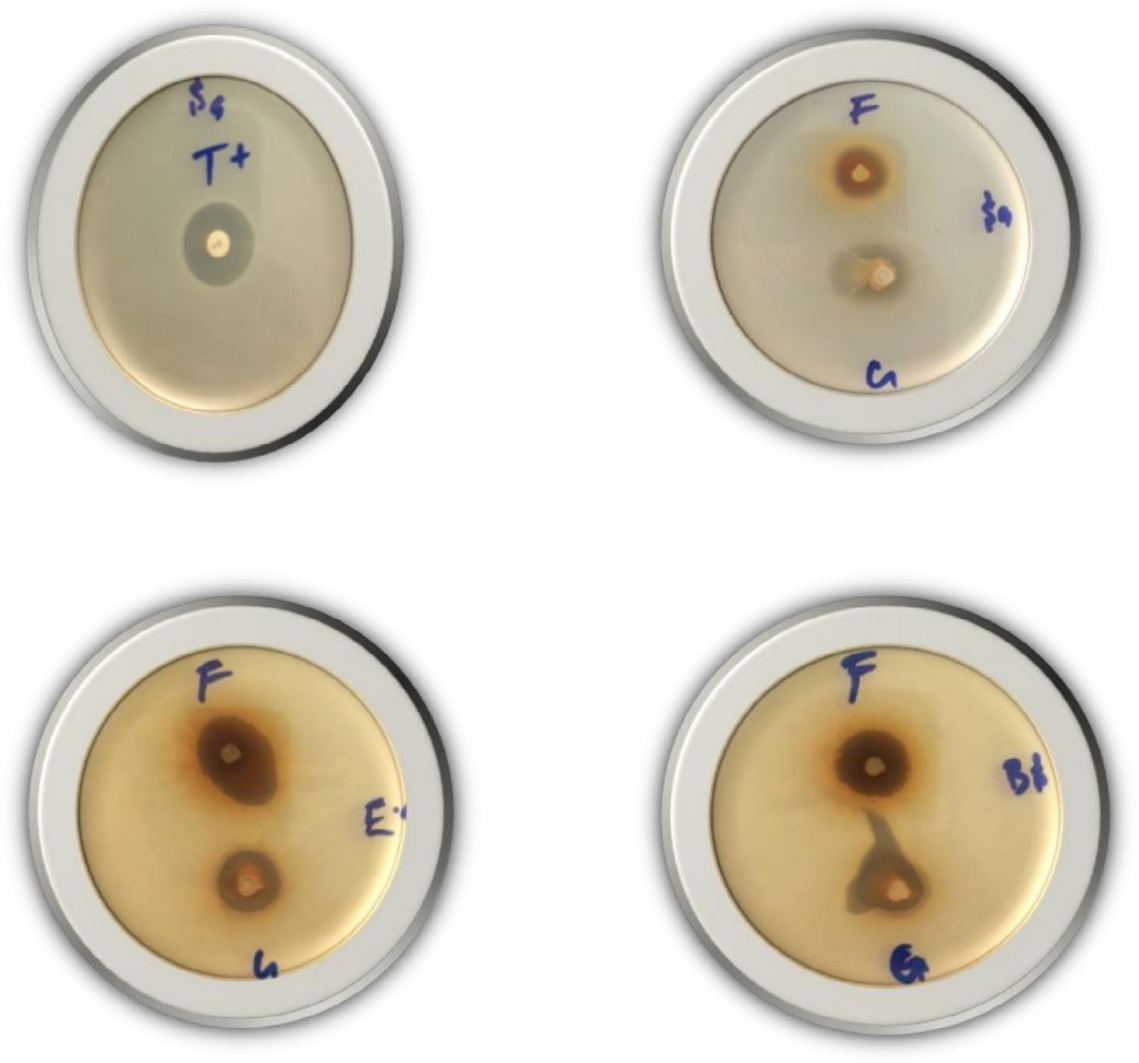
Antimicrobial properties of JPEF and JPEG studied on solid media.

The antimicrobial potency of the JPEF and JPEG studied was assessed on solid media to determine the growth inhibition diameter of the selected microbial strains (Figure 9). The evaluation of the antibacterial power of the leaf extract (JPEF) revealed that the largest inhibition diameter was recorded on *E. coli* with a diameter of approximately 20.36 mm, followed by *K. pneumoniae* with a diameter of approximately 17.58 mm (Table 3). However, a smaller inhibition diameter of 13.65 mm was observed on another strain. The fruit extract (JPEG) revealed a large inhibition diameter of 18.36 mm on *S. aureus* , followed by *P. mirabilis* with a diameter of 17.29 mm (Table 3). The positive control used (Amp) showed that *E. coli* and *K. pneumoniae* are resistant strains, while *S. aureus* and *P. mirabilis* are susceptible strains with inhibition diameters of 10.36 and 11.44 mm, respectively. The essential oil of *Juniperus* species was shown by Hajjouji et al. (2019) to be efficient against *E. coli* (13.23 mm) and *S. aureus* (31.12 mm). Numerous investigations have demonstrated that *S. aureus* is susceptible to the bioactive components of *Juniperus*, most notably the work of Mansouri et al. (2010). Additionally, Bahri et al. (2012) found that *Juniperus* essential oil had antibacterial action against *E. coli* (18.8 mm) and *S. aureus* (27 mm). It should be noted, however, that methodological differences (extract concentration, type of disc, culture medium, and incubation conditions) may influence the size of the inhibition zones. Nevertheless, in general, JPEF has comparable antibacterial potential.

**TABLE 3:** Antibacterial and antifungal activity of JPEF and JPEG, studied and positive controls.

	*E. coli*	*S. aureus*	*K. pneumoniae*	*P. mirabilis*	*S. cerevisiae*	*C. albicans*
JPEF	20.36 ± 2.50^c^	16.96 ± 1.14^c^	17.58 ± 0.86^c^	13.65 ± 1.20^b^	19.55 ± 1.42^c^	22.25 ± 0.85^c^
JPEG	16.84 ± 1.53^c^	18.36 ± 1.71^c^	16.74 ± 1.62^c^	17.29 ± 1.91^c^	18.47 ± 0.17^c^	20.38 ± 1.27^c^
Amp	0.0 ± 0.0^a^	10.36 ± 0.95^b^	0.0 ± 0.0^a^	11.44 ± 0.89^b^	—	—
Flu	—	—	—	—	14.58 ± 0.73^b^	13.59 ± 0.78^b^

*Note:* Different letters indicate significant differences (*p* < 0.05). JPEF: leaf extract; JPEG: fruit extract; Amp: ampicillin; Flu: fluconazole. Data are expressed as mean ± SD of inhibition zone diameters (mm).

Evaluation of the antifungal potency of JPEF and JPEG revealed a large diameter of inhibition on *C. albicans* , with diameters of around 22.25 and 20.38 mm (Table 3), respectively. On the other hand, JPEF and JPEG showed inhibition on *S. cerevisiae* , with diameters of around 19.55 and 18.47 mm, respectively. However, the positive control (Flu) showed that both strains were susceptible, with inhibition diameters of 14.58 mm for *S. cerevisiae* and 13.59 mm for *C. albicans* (Table 3).

Structural differences between bacteria considerably influence their sensitivity. Gram‐positive bacteria, lacking an outer membrane, have a more permeable cell wall, which disrupts proton motility, electron flow, active transport, and coagulation of cell contents (Ennajar et al. 2009). The hypersensitivity of the *S. aureus* strain could be attributed to the increased vulnerability of Gram‐positive bacteria to environmental variations, such as temperature, pH, and natural JPEF and JPEG, due to the absence of an external membrane (Balentine et al. 2006). In contrast, the growth of Gram‐negative bacteria can be inhibited by factors that disrupt cellular integrity and membrane permeability, such as low pH and high NaCl concentrations (Georgantelis et al. 2007).

Several classes of polyphenols, such as phenolic acids, flavonoids, and tannins, play a crucial role in plant defense mechanisms against pathogenic micro‐organisms, insects, and herbivores (Falleh et al. 2008). Notable antibacterial substances among these polyphenols include epigallocatechin, catechin, myricetin, quercetin (Shan et al. 2007), and luteolin (Askun et al. 2009).

According to Miceli et al. (2009), polyphenols, and flavonoids in particular, are effective antimicrobial agents against a variety of microorganisms. Research into the mechanism of action of flavonoids has revealed that these compounds do not act at a specific site but rather have multiple targets. For example, the activity of apigenin is partially attributed to the inhibition of DNA gyrase. The antimicrobial efficacy of JPEF and JPEG depends not only on the presence of phenolic compounds but also on other secondary metabolites, such as terpenoids, whose antimicrobial activity is widely recognized (Miceli et al. 2009). Falleh et al. (2008) add that this antimicrobial activity is also influenced by the position and number of hydroxyl groups.

### 3.6 In Silico Evaluation of Biological Activity

**FIGURE 10:**
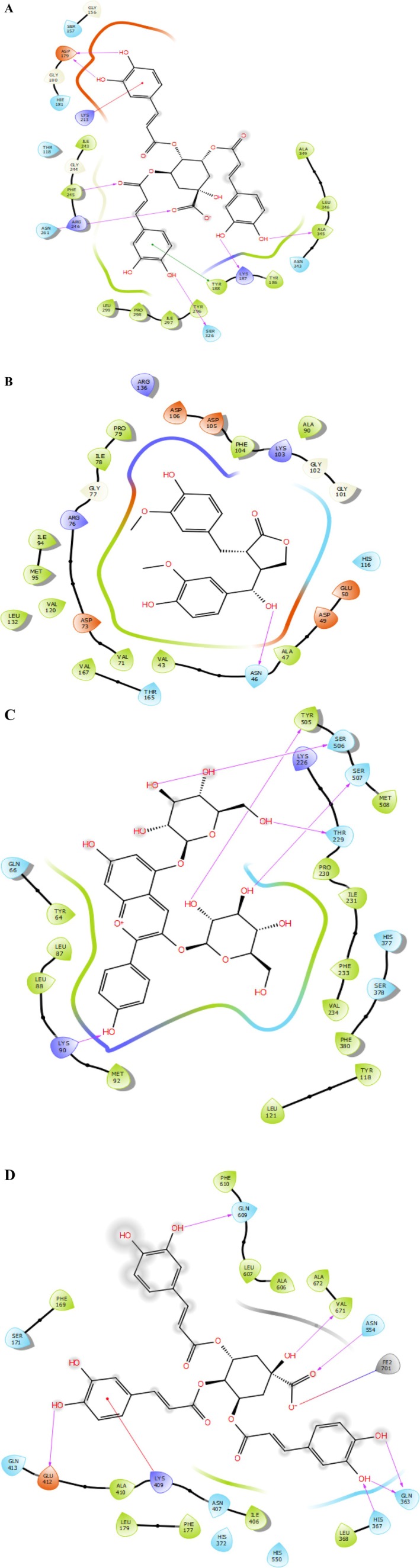
The 2D viewer of ligand interactions with the active site. (A, D): 4,5‐*O*‐dicaffeoylquinic acid interactions with the active site of NADPH oxidase and lipoxygenase. (B): 7‐Hydroxymatairesinol interactions with the active site of gyrase B. (C): Pelargonidin‐3,5‐*O*‐diglucoside interactions with the active site of ergosterol.

**FIGURE 11:**
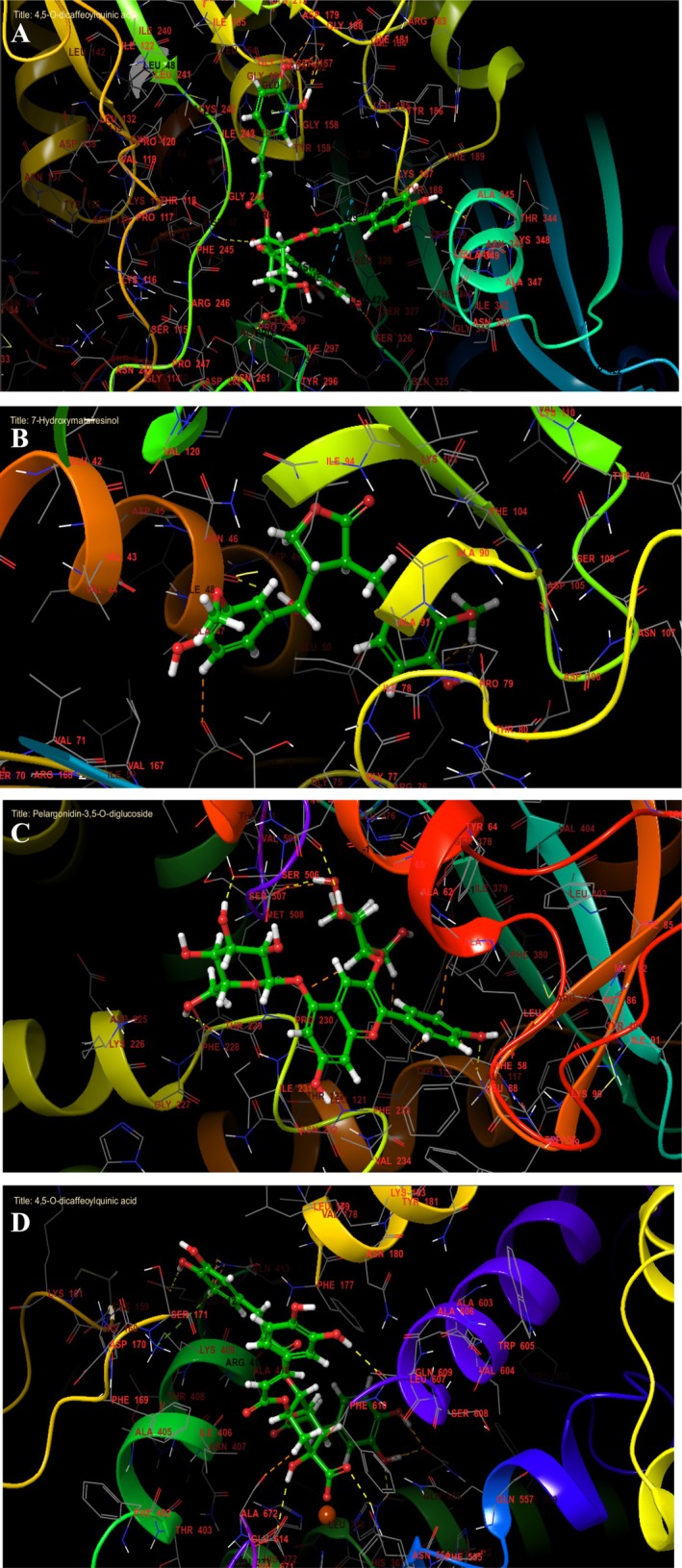
The 3D viewer of ligand interactions with the active site. (A, D): 4,5‐*O*‐dicaffeoylquinic acid interactions with the active site of NADPH oxidase and lipoxygenase. (B): 7‐Hydroxymatairesinol interactions with the active site of gyrase B. (C): Pelargonidin‐3,5‐*O*‐diglucoside interactions with the active site of ergosterol.

NADPH oxidase is a critical enzyme in reactive oxygen species (ROS) production, and its inhibition is a key strategy for mitigating oxidative stress (Figures 10 and 11). Among the tested compounds, 4,5‐O‐dicaffeoylquinic acid exhibited the strongest binding affinity with a Glide score of −7.124 kcal/mol, followed by Pelargonidin‐3,5‐O‐diglucoside (−6.975 kcal/mol) and Cyanidin‐3,5‐O‐diglucoside (−6.68 kcal/mol). These results suggest their potential as potent antioxidants by targeting NADPH oxidase activity. Gyrase B is essential for bacterial DNA replication, making it a prime target for antibacterial agents. The most active inhibitors were 7‐Hydroxymatairesinol (−7.89 kcal/mol), Cyanidin‐3,5‐O‐diglucoside (−6.837 kcal/mol), and Pelargonidin‐3,5‐O‐diglucoside (−6.722 kcal/mol) (Table 4). Their high binding affinities indicate strong interactions with the enzyme's active site, potentially disrupting DNA supercoiling and bacterial growth.

**TABLE 4:** Docking results in ligands in different receptors.

	Glide gscore (kcal/mol)			
	2CDU	3G7E	5FSA	3 V99
4,5‐O‐dicaffeoylquinic acid	−7.124	−5.226	−7.226	−8.429
7‐Hydroxymatairesinol	−5.776	−7.89	−8.059	−5.563
Cyanidin‐3,5‐O‐diglucoside	−6.68	−6.837	−9.041	−7.283
Dihydroferulic acid 4‐O‐glucuronide	−5.965	−4.824	−7.364	−7.323
Ferulic acid‐4‐O‐glucuronide	−5.626	−4.181	−5.742	−7.222
p‐Coumaric acid 4‐O‐glucoside	−5.376	−5.043	−5.868	−6.623
Pelargonidin‐3,5‐O‐diglucoside	−6.975	−6.722	−9.31	−7.268
Procyanidin dimer B1	−5.369	−4.643	−4.973	−6.753

Ergosterol is a vital component of fungal cell membranes. The top‐performing compounds were Pelargonidin‐3,5‐O‐diglucoside (−9.31 kcal/mol), Cyanidin‐3,5‐O‐diglucoside (−9.041 kcal/mol), and 7‐Hydroxymatairesinol (−8.059 kcal/mol) (Table 4). These molecules demonstrated significant binding affinity, suggesting their ability to interfere with ergosterol biosynthesis or membrane integrity. Lipoxygenase plays a crucial role in the synthesis of inflammatory mediators. The most promising inhibitors were 4,5‐O‐dicaffeoylquinic acid (−8.429 kcal/mol), Pelargonidin‐3,5‐O‐diglucoside (−7.268 kcal/mol), and Cyanidin‐3,5‐O‐diglucoside (−7.283 kcal/mol). Their strong binding interactions highlight their potential as anti‐inflammatory agents by modulating lipoxygenase activity.

The 2D and 3D interaction analysis revealed that 4,5‐O‐dicaffeoylquinic acid established several key contacts within the active site of NADPH oxidase (PDB ID: 2CDU). Specifically, the ligand formed seven hydrogen bonds with residues ASP 179, PHE 245, ASN 261, SER 326, LYS 187, and ALA 315, and one Pi cation with residue LYS 213, and one Pi‐Pi stacking with residue TYR 188. These interactions likely contribute to stabilizing the ligand within the binding pocket, enhancing its affinity and potential modulatory activity (Figures 10A and 11A).

Concerning the antimicrobial activity, in the active site of gyrase B (PDB ID: 3G7E), 7‐Hydroxymatairesinol established one hydrogen bond with residue ASN 46 (Figures 10B and 11B), while Pelargonidin‐3,5‐O‐diglucoside established five hydrogen bonds with residues TYR 505, SER 506, SER 507, THR 229, and LYS 90 (Figures 10C and 11C).

Furthermore, 4,5‐O‐dicaffeoylquinic acid established seven hydrogen bonds with residues GLN 609, VAL 671, ASN 554, GLN 363, HIS 367, and GLU 412, one Pi cation with residue LYS 409, and one salt bridge with residue FE2 701 in the active site of lipoxygenase (Figures 10D and 11D).

Although the *in silico* molecular docking results provide a valuable predictive framework for the multi‐pharmacological potential of *J. phoenicea* ssp. *Turbinata* phytoconstituents, several inherent technical limitations must be acknowledged to ensure a balanced interpretation of the data. First, the use of a static or semi‐flexible receptor model represents a significant constraint, as it fails to capture the essential time‐dependent conformational fluctuations and “induced fit” or “conformational selection” dynamics that characterize real‐time protein‐ligand recognition in biological systems (Antunes et al. 2015; Armen et al. 2009; Gioia et al. 2017). Second, the oversimplified treatment of solvation and desolvation effects, specifically the neglect of explicit water‐mediated hydrogen bond networks and the energetic penalty associated with displacing water molecules from the binding pocket, can lead to inaccuracies in the estimation of binding free energies (Pavlovicz et al. 2020; Roberts and Mancera 2008; Van Dijk and Bonvin 2006). Third, reliance on empirical or force‐field‐based scoring functions imposes additional constraints, as these functions often struggle to model long‐range electrostatic interactions, polarization effects, and the complex entropic contributions to binding affinity (Li et al. 2025; Pachiappan et al. 2025). Consequently, while docking is an efficient tool for hypothesis generation and pose prediction, the absolute scores may not always perfectly correlate with experimental inhibitory concentrations (Lam and Katritch 2025). Therefore, to establish definitive biological relevance, these computational findings must be considered preliminary and necessitate rigorous future validation through Molecular Dynamics (MD) simulations, mechanistic in vitro enzyme assays, and in vivo pharmacological studies (Hasan et al. 2025; Kotev et al. 2016).

## 4 Conclusion

The in vivo experiments demonstrated that JPEF and JPEG are non‐toxic at doses of 200 and 400 mg/kg and exhibit marked anti‐inflammatory and analgesic activities. Complementary in vitro assays further revealed strong antioxidant, antibacterial, and antifungal properties. These biological activities are consistent with the high phenolic content of the JPEF and JPEG, as confirmed by LC–MS/MS analysis. Additionally, molecular docking studies suggest possible interactions of the major identified compounds with targets such as NADPH oxidase, COX, LOX, and DNA gyrase, providing mechanistic insights that may underlie the observed biological effects. Collectively, these findings highlight the substantial pharmacological potential of *J. phoenicea* ssp. *Turbinata* and support its traditional use as a medicinal plant. Nevertheless, additional studies are required to identify the specific bioactive constituents responsible for these effects and to clarify their underlying mechanisms of action. Future work should also examine possible synergistic interactions among phenolic compounds and evaluate the JPEF and JPEG in more complex biological and pathological models. Advancing this research could ultimately contribute to the development of novel natural therapeutic agents derived from *J. phoenicea* .

## Author Contributions

**Musaab Dauelbait:** writing – original draft, writing – review and editing. **Tarik Chelouati:** writing – original draft, writing – review and editing, conceptualization. **Abdelfattah E. L. Moussaoui:** writing – review and editing, writing – original draft. **Salah‐eddine Chebaibi:** data curation, conceptualization. **Esmael M. Alyami:** supervision, writing – review and editing. **Azeddin El Barnossi:** methodology, software. **Yousef A. Bin Jardan:** data curation, formal analysis. **Amira Metouekel:** formal analysis, writing – review and editing. **Mostafa Messkini:** writing – original draft, formal analysis. **Ohoud A. Alghamdi:** supervision, writing – review and editing. **Soufyane Lafraxo:** methodology, writing – original draft, data curation. **Musa A. Said:** writing – review and editing, validation. **Ahmed Samir Benjelloun:** conceptualization, methodology. **Gehan M. Elossaily:** writing – review and editing, investigation.

## Funding

The authors extend their appreciation to the Deanship of Scientific Research and Graduate Studies at King Khalid University for funding this work through the Large Research Project under grant number RGP2/40/47 and AlMaarefa University through research under project number MHIRSP2025‐018.

## Disclosure

Plant Collection Approval: An approval was obtained from the Moroccan authority to collect *J. phoenicea* ssp. *turbinata* for research purposes. IUCN Policy Statement: The collection of plant material complies with relevant institutional, national, and international guidelines and legislation.

## Ethics Statement

The Ethical Committee of the Dhar Mahraz, Sidi Mohamed Ben Abdellah University, 30,000 Fez, Morocco, reviewed and approved this study. Notably, all methods adhered to the ethical guidelines outlined in the “Guide for the Care and Use of Laboratory Animals” published by the National Academy of Sciences.

## Consent

The authors have nothing to report.

## Conflicts of Interest

The authors declare no conflicts of interest.

## Data Availability

All data generated or analyzed during this study are included in this article.
